# Institutional Notes and News

**Published:** 1910-05-07

**Authors:** 


### Institutional Notes and News.

**Surrey Hospital, Guildford.**

No one can regard the position of affairs at the Royal Surrey County Hospital as otherwise than serious. This is not so much, perhaps, because of its financial straits as because of the increasing applications for admission, many of which at present have to be refused. Last year's income was £6,065 and the expenditure £6,134, leaving a manageable discrepancy of £69. But it is not these £69, it is the fifty odd patients waiting for admission who are the gravest fact in the case. When it is remembered that 1,078 in-patients were treated last year as it was, it is clear that further accommodation is necessary. Fortunately, we understand, this need not mean immediate building, except perhaps for more servants' quarters, for there is a spare ward which has been kept furnished enough for an emergency, and which could be opened permanently if the maintenance money were raised. The sum needed for this purpose is £1,000 if the full tale of beds is to be used. The public therefore has to decide whether it will subscribe this money or whether it will evade—for a time—its responsibility by forcing the sale of the hospital's invested funds. Dr. Chester, at the annual meeting, said he very much hoped that the new ward would be opened and set apart for paying patients. He also made a good point in reminding the meeting that competition was very great to-day for bequests and donations between all kinds of institutions, and if they once disturbed the confidence of the public in the security of their bequests they would have people declining to leave them legacies. A special fund has been started for the upkeep of the ward, for which we understand about £1,000 has been raised already. Some land opposite the hospital is for sale, of which the hospital has the first refusal. It will be decided before Michaelmas whether this open space is to be retained, or whether the County Education Authority is to become possessor of it.

**M. Cambon at the French Hospital.**

M. Cambon, the French Ambassador, presided over the Forty-second Annual Dinner in aid of the French Hospital, at the Hotel Cecil last Saturday. The following is an abstract of his speech in proposing "The Founders and Benefactors of the Hospital": "I am pleased to see present so many members of the Diplomatic Body and representatives of the City. France was greatly touched by the generosity of the City at the time of the Paris floods. The fund opened at the Mansion House under the patronage of the Lord Mayor and its unexpected result could not fail to make my countrymen grateful for the sympathy exhibited towards them. Paris has always regarded the City of London as its best friend and thanks the citizens for their support of French institutions in London. With regard to the work of the hospital, it now includes nineteen wards containing together seventy-four beds, an operating theatre, two consultation rooms, and a dispensary. The admissions since the foundation to December 31 last numbered 21,254 in-patients and 441,599 out-patients of every nationality." We are glad to add that during the evening subscriptions totalling £3,700 were announced. The hospital was opened in 1867 for the benefit of all foreigners in need of medical treatment, and eventually it coalesced with the French Dispensary that Dr. Achille Vintras had started six years earlier. As the French Ambassador remarked, a distinguished company was present, among whom were Mr. E. Lazarus, President of the Hospitals Committee; Mr. A. Baume, Hon. Secretary; M. Pondepeyre, Secretary; and M. Phalempin, Hon. Treasurer.

**Coventry and Warwickshire Hospital.**

A serious state of affairs was made only too apparent in the report of the House Committee at Coventry for the five weeks ending on April 13. Briefly, this report showed that the hospital at present is crowded to the limit of its powers, an average of between forty and fifty patients is waiting admission, and, in the words of Mr. Harrison Butler, "cases are being treated as out-patients, which in nine out of ten hospitals in the country would be treated as in-patients." This clearly is a condition of urgency which prompt steps must be taken to meet, and it has been resolved to ask the Mayor to call a meeting of the town to consider the matter; meanwhile the House Committee has exercised its authority by obtaining estimates and plans for the extension scheme, which has already been the subject of discussion. The plans have been prepared by Mr. Chattaway, and are awaiting the criticism of the Medical Committee. A glance at the figures presented by the House Committee shows that, as Mr. Alfred Herbert, the chairman, said, "a stream of patients" is passing through the wards, and it is not because of their length of stay that the hospital is congested. The actual figures for the period reported on were: First week, daily average 77.5, and waiting admission 48; second week, 76.7 and 39; third week, 74.7 and 35; fourth week, 79.2 and 41; fifth week, 79.5 and 38. At the time of the meeting 49 patients were waiting admission. As regards finances, the hospital's income is behindhand, but, as Mr. Alfred Herbert pointed out, the increase of £58 in their expenditure was reasonably accounted for by the stress of work they were undergoing.

**The King Edward VII. Sanatorium.**

The Third Annual Report of the King Edward VII. Sanatorium, Midhurst, has some very interesting facts and additions, including the climatological observations of the sanatorium as a health station. The beds have been fully occupied, but the medical staff rightly has one regret, which is the relatively small proportion of cases (only 25.7 per cent.) which apply for admission while still in an early stage of disease. The number of patients discharged during the year was 292, and the report divides these into three groups, incipient, moderate, and advanced; the examination previous to admission, which was started a year ago, is hoped to reduce the numbers of patients in group three and to increase the numbers in the first two groups. Work in the grounds for patients is continued with useful results, group one providing, naturally, the largest percentage of workers—namely seventy-five. The Report goes on to give a very careful analysis of the groups for clinical purposes, and has the important addition of "after histories" of patients discharged since July 1906. A few examples are given of the reports which under this heading former patients have sent in. Statistics of the ultimate results, and a laboratory report containing, in the words of the Preface, "scientific researches of a very high order on the various vaccines of streptococci and staphylococci, on the temperature and opsonic index of cases of pulmonary tuberculosis" then follow, and the volume—for that is what its seventy-two pages of letterpress and charts deserve to be called—comes to an end with the climatological section. The volume is capitally printed and produced, and will interest every reader of either section of The Hospital. Such a compilation, however, surely deserves a list of contents; its absence is the one fault on which criticism can fasten.

